# User Interaction Modeling and Profile Extraction in Interactive Systems: A Groupware Application Case Study †

**DOI:** 10.3390/s17071669

**Published:** 2017-07-20

**Authors:** Cristina Tîrnăucă, Rafael Duque, José L. Montaña

**Affiliations:** Departamento de Matemáticas, Estadística y Computación, Universidad de Cantabria, 39005 Santander, Spain; rafael.duque@unican.es (R.D.); joseluis.montana@unican.es (J.L.M.)

**Keywords:** human–computer interaction, user modeling, mobile interfaces, weighted automaton, clustering

## Abstract

A relevant goal in human–computer interaction is to produce applications that are easy to use and well-adjusted to their users’ needs. To address this problem it is important to know how users interact with the system. This work constitutes a methodological contribution capable of identifying the context of use in which users perform interactions with a groupware application (synchronous or asynchronous) and provides, using machine learning techniques, generative models of how users behave. Additionally, these models are transformed into a text that describes in natural language the main characteristics of the interaction of the users with the system.

## 1. Introduction

At present, a proliferation of groupware applications (social networks, shared editors, messaging services, etc.) can be observed, enabling collaboration between users The computer-supported collaborative work (CSCW) research field [1] has focused on studying how technology can effectively support these collective processes. One of the main purposes of the CSCW has been the identification of methodologies that provide a systematic approach for discovering the users’ requirements, and for evaluating the degree to which these systems allow users to be aware of, modify or interact with the work of other users.

One of the most frequent characterizations of the groupware systems is based on the following two dimensions of their contexts of use [2]: (1) *time*, whether users collaborate synchronously (they collaborate at the same time) or asynchronously (they collaborate at different moments); and (2) *space*, whether users are co-located or geographically distributed. Currently, most users of smart-phones use groupware applications that allow them to collaborate synchronously and/or asynchronously while being geographically distributed. Therefore, the evaluation of these groupware applications should assess the versatility of the systems to support synchronous and asynchronous interactions and whether the system is adapted to the users’ mental model so they do not have to make any effort to use the system’s features.

**Figure 1:**
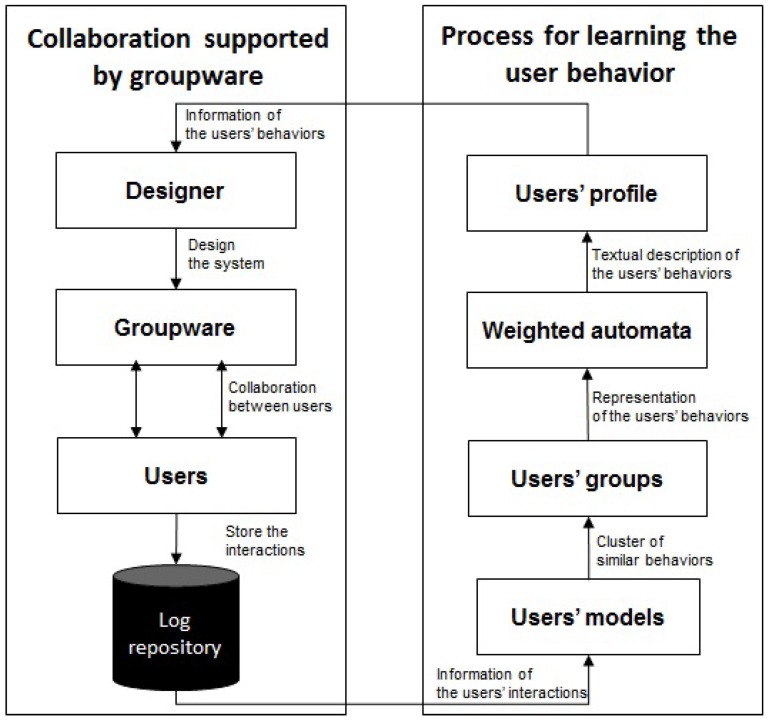
Main steps of the methodology.

In order to carry out these evaluations, this article describes a methodological approach (see Figure 1) that identifies the context of use in which the users perform the interactions (synchronous or asynchronous) and generates descriptive models of how these users orchestrate the interactions with the system. Finally, these models are processed to create a descriptive text in natural language of the main characteristics of the users’ interaction with the system. Thus, the designer can verify whether the system offers a natural interaction experience to its users in each context of use and whether users deviate substantially from the task model used to design the system. The task models are specifications widely used in the human–computer interaction (HCI) field to describe the logical activities that have to be performed in order to reach the users’ goals. Therefore, the evaluation of the system should analyze the users’ behaviors to verify that it is not hard for them to follow the sequence of actions specified by the task model. Moreover, the users of the system can have different behaviors so the task model must also be flexible enough to enable the users to reach their goals in different ways. With this aim, we propose a methodology that processes log repositories with information of the user interactions and also models the behavior of the users. Then, the users with similar behaviors are grouped together and for each set of similar users, a profile (in the form of a weighted automaton) is generated. Finally, these weighted automata are transformed into descriptive text that capture the characteristics of those different user profiles, which is valuable information that allows for the design of a task model according to the users’ natural behavior. With respect to our previous work [3], we introduce two new models for the users’ behavior, one of them allowing for more flexibility (the user is no longer represented by a fixed-length vector), and we highlight the pros and cons of using any of the three proposed models. The experimental section is modified accordingly.

Note that user modeling can be understood in a more general context (from an artificial intelligence perspective), as the process through which systems obtain information about the individual characteristics of users [4,5]. On the other hand, in the HCI field, a lot of work has been done on ontology-based models, where three aspects are considered: user, context and device [6,7,8,9] (we refer the reader to [10] for a very good chronological review of the evolution of user models).

## 2. Related Work

Suarez et al. [11] establish a classification of interactive systems and point out that specific evaluation criteria should be applied to each one, according to their features and complexity. This classification includes the groupware as a specific type of application. Among the specific features of the groupware applications, it is noteworthy that they enable not only the communication between users but also the collaborative building of artifacts in a shared workspace. The groupware applications support user interactions whose effects can be perceived by at least another member of the group or another community. Therefore, the evaluation should consider specific criteria such as the effectivity of the awareness and social support of the application. Molina et al. [12] propose a combination of techniques (questionnaires, empirical testing, heuristic evaluation, eye tracking) to analyze the awareness support of groupware applications. Neale et al. [13] propose some specific measurements to evaluate the degree to which an application intuitively enables social interactions.

These evaluations have been automated using tools such as Tatiana, which is independent of any groupware system and allows us to configure and to automate the analysis of interactions recorded in log files by means of several socio-cognitive methodologies. Moreover, Tatiana provides support to perform non-automated analysis, where collaborative activity is reproduced by video and the users introduce annotations or categorizations about the users’ interactions. ProM [14] is another tool that facilitates this kind of analysis: it allows users to select different data mining algorithms in order to analyze the work-flows recorded in log files.

Thaler [15] uses the term *usability mining* for the evaluation process of information systems based on analyzing log traces. In this case, the goal is to derive a model of usage of the system that includes information about the users behavior (irrelevant actions, undo actions, using help function, etc.). The users’ actions can be analyzed using statistical techniques that generate quantitative information. These measures are known as low-level indicators [16], as they usually do not provide an interpretation of the user’s activity. The indicators whose values provide an interpretation (cognitive, technological, etc.) of the user’s activity are known as high-level indicators.

## 3. Data Flow Representation

The data flow generated by the interactions of users with a computerized system can be seen as a stream of tuples D of the form (id,t,a), where id is the user’s identifier in the system and *t* represents the instant of time when the action *a* took place.

More precisely, D is a sequence of the form ${\left[\left(id_{i},t_{i},a_{i}\right)\right]}_{1\leq i\leq N}$ where each $\left(id_{i},t_{i},a_{i}\right)$ belongs to U×T×A, U is a finite set of user identifiers, T is a finite set of instants in which the process is observed and A is a finite set of actions. We assume that $t_{i}\leq t_{i+1}$, for 1≤i<N−1. The information contained in D can be segmented according to different criteria. If we are interested in the case in which D is partitioned according to the user identifiers, $U:=\{u_{1},...,u_{l}\}$, each segment of information is of the form:

$$D\left(k\right):=\left[\left(a_{i},t_{i}\right):\;id_{i}=u_{k}\right]=\left[\left(\;a_{i_{1}},t_{i_{1}}\right),\left(a_{i_{2}},t_{i_{2}}\right),\ldots ,\left(a_{i_{N_{k}}},t_{i_{N_{k}}}\right)\right]$$

That is, segment D(k) is the subsequence of D formed by those tuples $\left(a_{i},t_{i}\right)$ in which action $a_{i}$ is performed by the user whose identifier is $u_{k}$ (the user’s id information is the same for all tuples in D(k) and thus it is omitted). We call segment D(k) a trace (also *path* or *trajectory*) of user $u_{k}$ in the system.

For data analysis and user profiling, one might be interested in the duration of transitions between actions and not in the exact instance of time in which actions take place. In such cases, we can rewrite the trace D(k) as a new path of the form:

(1) $$\pi \left(k\right):=[\left(a_{i_{1}},d_{i_{1}}\right),\ldots ,\left(a_{i_{N_{k}-1}},d_{i_{N_{k}-1}}\right),\left(a_{i_{N_{k}}},\right)]$$

where $d_{i_{j}}:=t_{i_{j+1}}-t_{i_{j}}$ is the time elapsed between action $a_{i_{j}}$ and action $a_{i_{j+1}}$, for $1\leq j<N_{k}$, and thus is a continuous attribute. We can discretize these durations into a fixed number of categories, say $C:\;=\;\{c_{1},\ldots ,c_{L}\}$, where each value $c_{i}$ represents a time interval chosen ad hoc for the system under study.

## 4. User Interaction Models

The problem of *modeling the user interactions* can be approached from two very different perspectives. One possibility is to identify each user with its trajectory and determine which users behave similarly using a path-similarity measure. The other option is to find a generative model that behaves in the same way as the user does in the interactive system. A priori there is no restriction on the nature of the generative model, which could be a Markov model, an automaton, a Bayesian network, etc. We explore here a Markov-like structure called *weighted automaton*.

**Definition** **1.** *Weighted automaton (see [17]). Let* Σ *be a finite alphabet of symbols and *n* a positive natural number. A weighted automaton over* Σ *with n states is a tuple* $M:=(in,out,{\left\{W_{\sigma }\right\}}_{\sigma \in \Sigma })$*, where* in *and* out *are vectors in* $R^{n}$ *representing features of the empty prefix and of the empty suffix, respectively, and* $W_{\sigma }$ *is an* n×n*-matrix with real entries representing transition weights. In some situations we can omit* in *and* out *if they are not relevant for the problem.*

Let A be the set of actions that can be performed by a user in a given interactive system. For each user $u_{k}$, we build the path π(k) as in (1). In this path, actions $a_{i_{j}}$ are in A and durations $d_{i_{j}}$ and belong to some interval *c* in C, where $C=\{c_{1},\ldots ,c_{L}\}$ is a finite set of time intervals as explained in Section 3. We define the weighted automaton $M_{u_{k}}:=\left({\left\{W_{c}^{k}\right\}}_{c\in C}\right)$ over the alphabet C with M=|A| states, in which the matrices of weights are defined as follows:

$$W_{c}^{k}\left(a,a^{\prime }\right):=count_{c}\left(a,a^{\prime }\right),\;\mathrm{for}\mathrm{all}c\in C\;\mathrm{and}\;a,a^{\prime }\in A$$

where $count_{c}\left(a,a^{\prime }\right):=\left|\left\{j\in \left\{1,\ldots ,N_{k}-1\right\}|a=a_{i_{j}},a^{\prime }=a_{i_{j+1}},d_{i_{j}}\in c\right\}\right|$, that is, the number of times action *a* precedes action $a^{\prime }$ in the trajectory π(k) of the respective user and the time elapsed between the two actions is in the time category *c*.

Depending on the application under study, we may be interested in the sequentiality of the user’s actions (see *Model a* below) or in the frequency with which each action is performed (*Model b*). Alternatively, the user’s behavior can be described by its path, which also leads to further categorization based on the number of previous actions we consider (*Model 0*, *Model 1*, *Model 2*, etc.).

- *Model a*: we identify the user $u_{k}$ with a point of the affine space $R^{LM^{2}}$ defined by,
  (2) $$A_{u_{k}}:={\left(W_{c}^{k}\left(a,a^{\prime }\right)\right)}_{(c,a,a^{\prime })\in C\times A\times A}\in R^{LM^{2}}$$
- *Model b*: the behavior of user $u_{k}$ is a point of the affine space $R^{LM}$ defined by,
  (3) $$B_{u_{k}}:={\left(\sum_{a^{\prime }\in A}W_{c}^{k}\left(a,a^{\prime }\right)\right)}_{(c,a)\in C\times A}\in R^{LM}$$
- *Model p*, p≥0: we identify the user $u_{k}$ with a trace of variable length defined by,
  $$\pi^{p}\left(k\right):=\left[\left(a_{i_{1}},c_{i_{1}},\ldots ,a_{i_{p+1}},c_{i_{p+1}}\right),\left(a_{i_{2}},c_{i_{2}},\ldots ,a_{i_{p+2}},c_{i_{p+2}}\right),\ldots ,\left(a_{i_{n-1-p}},c_{i_{n-1-p}},\ldots ,a_{i_{n-1}},c_{i_{n-1}}\right)\right],$$
  where $c_{i_{j}}$ is the time interval to which $d_{i_{j}}$ belongs and p≥0. In particular, if p=0, we have:
  $$\pi^{0}\left(k\right):=\left[\left(a_{i_{1}},c_{i_{1}}\right),\ldots ,\left(a_{i_{n-1}},c_{i_{n-1}}\right)\right].$$

Determining whether two users have a similar behavior can then be done via distance measures or similarity measures. If users are represented as paths of variable length, their similarity can be calculated using a Monte Carlo estimation of the crossed entropy between their respective traces (see Equation (7) in [18] or Equation (11) in [19]).

(4) $$H_{MC}\left(\pi^{p}\left(k_{1}\right),\pi^{p}\left(k_{2}\right)\right):=-\frac{1}{n}\sum_{i=1}^{n}log\left[\frac{\sum_{j=1}^{m}I_{\left\{o_{j}^{\prime }\right\}}\left(o_{i}\right)}{m}\right]$$

where $\pi^{p}\left(k_{1}\right)=\left[o_{1},\ldots ,o_{n}\right],\pi^{p}\left(k_{2}\right)=\left[o_{1}^{\prime },\ldots ,o_{m}^{\prime }\right]$ and $I_{\left\{o_{j}^{\prime }\right\}}$ is the indicator function of the set $\left\{o_{j}^{\prime }\right\}$.

Moreover, to improve estimations when the available traces are too short and do not cover the entire set of available actions (along with their respective time intervals), we can use Laplace smoothing, and modify the equation as follows (see Equation (8) in [18] or Equation (12) in [19]).

(5) $$H_{MC}\left(\pi^{p}\left(k_{1}\right),\pi^{p}\left(k_{2}\right)\right):=-\frac{1}{n}\sum_{i=1}^{n}log\left[\frac{\sum_{j=1}^{m}I_{\left\{o_{j}^{\prime }\right\}}\left(o_{i}\right)+1}{m+\left(|A\right|\times {\left|C|\right)}^{p+1}}\right]$$

The more similar traces are the smaller values of $H_{MC}$ we obtain, but the zero can only be reached when both traces consist of a constant number of identical elements. For this reason, $H_{MC}$ cannot be considered a distance function. Also, note that in general $H_{MC}\left(\pi^{p}\left(k_{1}\right),\pi^{p}\left(k_{2}\right)\right)$ and $H_{MC}\left(\pi^{p}\left(k_{2}\right),\pi^{p}\left(k_{1}\right)\right)$ are not equal. Nevertheless, this can be fixed by using the following formula.

(6) $$d_{MC}\left(\pi^{p}\left(k_{1}\right),\pi^{p}\left(k_{2}\right)\right):=\frac{H_{MC}\left(\pi^{p}\left(k_{1}\right),\pi^{p}\left(k_{2}\right)\right)+H_{MC}\left(\pi^{p}\left(k_{2}\right),\pi^{p}\left(k_{1}\right)\right)}{2}$$

For users represented as vectors of fixed length $x=(x_{1},\ldots ,x_{p})$ and $y=(y_{1},\ldots ,y_{p})$, the distance between them can be calculated using the *Minkowski metric*:

(7) $$d_{g}\left(x,y\right):=\left(\right|x_{1}-y_{1}{|}^{g}+|x_{2}-y_{2}{|}^{g}+\ldots +|x_{p}-y_{p}{{|}^{g})}^{1/g}$$

The commonly used *Euclidean distance* is obtained for g=2, the *Manhattan distance* (also called *city block*) for g=1 and the *Chebyshev distance* for g=∞.

An alternative concept to that of the distance is the similarity function. When the angle between the two vectors is a meaningful measure, one may consider the *cosine measure*,

(8) $$cos\left(x,y\right):=\frac{\langle x,y\rangle }{∥x∥\cdot ∥y∥},$$

where 〈·,·〉 is the Euclidean inner product in $R^{p}$, and $∥\cdot ∥$ is the norm induced by the inner product, or the *normalized Pearson correlation*:

(9) $$cor\left(x,y\right):=\frac{\langle x-\bar{x},y-\bar{y}\rangle }{∥x-\bar{x}∥\cdot ∥y-\bar{y}∥},$$

where $\bar{x}$ denotes the average feature value of *x* over all dimensions.

In practice, we use $d_{cos}\left(x,y\right):=1-cos\left(x,y\right)$ and $d_{cor}\left(x,y\right):=1-cor\left(x,y\right)$ in order to have $d_{cos}\left(x,x\right)=d_{cor}\left(x,x\right)=0$ as in the case of the above mentioned distances.

Note that each of the distances introduced so far corresponds to different goals, and choosing one of them should be made accordingly. In the sequel, we outline their differences using an oversimplified example in which we have only two possible actions (M=2 and $A=\{a_{1},a_{2}$) and we consider only one time interval (L=1 and $C=\{c_{1}\}$).

**Example** **1.** *Let $\pi^{0}\left(1\right)=\left[a_{1},a_{2},a_{1},a_{2}\right]$, $\pi^{0}\left(2\right)=\left[a_{1},a_{2},\ldots ,a_{1},a_{2}\right]$ and $\pi^{0}\left(3\right)=\left[a_{1},a_{2},a_{1},a_{1}\right]$ be the traces of three different users, of length 4, 40 and 4, respectively (the time interval is dropped for better readability).*

**Model a****Model b****Model 0**
**Model a****Model b****Model 0**$u_{1}$*(0, 2, 1, 0)**(2, 2)*$\pi^{0}\left(1\right)$$u_{1}$*(0, 2, 1, 0)**(2, 2)*$\pi^{0}\left(1\right)$$u_{2}$*(0, 20, 19, 0)**(20, 20)*$\pi^{0}\left(2\right)$$u_{3}$*(1, 1, 1, 0)**(3, 1)*$\pi^{0}\left(3\right)$$d_{1}$*36.000**36.000*-$d_{1}$*2.000**2.000*-$d_{2}$*25.456**25.456*-$d_{2}$*1.414**1.414*-$d_{\infty }$*18.000**18.000*-$d_{\infty }$*1.000**1.000*-$d_{cos}$*0.044**0*-$d_{cos}$*0.225**0.106*-$d_{cor}$*0.081**nan*-$d_{cor}$*0.478**nan*-$d_{MC}$--*0.693*$d_{MC}$--*0.752*

*Based on the Minkowski metrics, user 3 is much more similar than user 2 to user 1 in both* Model a *and* Model b, *while the metrics that measure the angle between vectors show user 2 to be much more similar than user 3 to user 1 (the equality of* Model a *and* Model b *values for the Minkowski metrics are fortuitous). The Monte Carlo-like estimation also marks user 2 as being more similar than user 3 to user 1. Therefore, if one wants to group together those users that perform actions in a similar fashion ignoring the length of their traces, then Minkowski metrics should be avoided. In contrast, these metrics are highly recommended when the length of the trace is an important user behavior aspect for the application under study.*

## 5. User Interaction Group Profiles

Next, we are interested in clustering the users into a certain non-specified quantity of *representative user profiles* such that users in the same group (cluster) behave more similarly to each other than to users in other clusters. To this end, we use agglomerative hierarchical clustering with three linkage criteria: single, complete and average (see [20] for more details). Given a set of *l* users to be clustered and a fixed number k≤l of desired clusters, the basic process of our hierarchical clustering is as follows.

- Step 1. Start by assigning each user to its own cluster, so that if we have *l* users, in this initial stage we have *l* clusters, each containing just one user.
- Step 2. Set the distances between the clusters equal the distances between the users they contain.
- Step 3. Find the closest (most similar) pair of clusters and merge them into a single cluster, so that now we have one less cluster.
- Step 4. Compute distances between the new cluster and each of the old clusters.
- Step 5. Repeat steps third and fourth until users are clustered into *k* clusters.

Depending on the type of linkage chosen, the distance between two clusters is computed with one of the three formulas:

- single linkage: $d\left(G,G^{\prime }\right)=\min_{x\in G,x^{\prime }\in G^{\prime }}d(x,x^{\prime })$
- complete linkage: $d\left(G,G^{\prime }\right)=\max_{x\in G,x^{\prime }\in G^{\prime }}d(x,x^{\prime })$
- average linkage: $d\left(G,G^{\prime }\right)=\left(\sum_{x\in G,x^{\prime }\in G^{\prime }}d(x,x^{\prime })\right)/(|G|\cdot |G^{\prime }\left|\right)$

where d(x,y) can be any of the distances previously defined.

We denote by *k* the number of representative user profiles in an interactive system. The correct choice of *k* depends most of the time on the application. The optimal *k* will strike a balance between maximum compression of user profiles using a single cluster, and maximum accuracy by assigning each profile to its own cluster (having one cluster per user). If an appropriate value of *k* is not apparent from prior knowledge on the properties of the profile set, it must be somehow determined. In the literature, there are several proposals for making this decision effective (see [20] for a survey). One popular proposal is to use the so called *elbow method*. The intuition behind this method is that one should choose *k* such that adding another cluster does not give much better modeling of the data.

However, because of the myriad of different settings (users represented as traces or as vectors, different distance metrics, different number of time intervals, different linkage criteria, etc.), we face a supplementary complication in determining the best *k* (see Example 2).

**Example** **2.** *Let us consider the following users (represented as vectors):*

$$u_{1}=\left(1,2\right),u_{2}=\left(1,3\right),u_{3}=\left(3,1\right),u_{4}=\left(5,4\right),u_{5}=\left(6,3\right).$$

**Figure 2:**
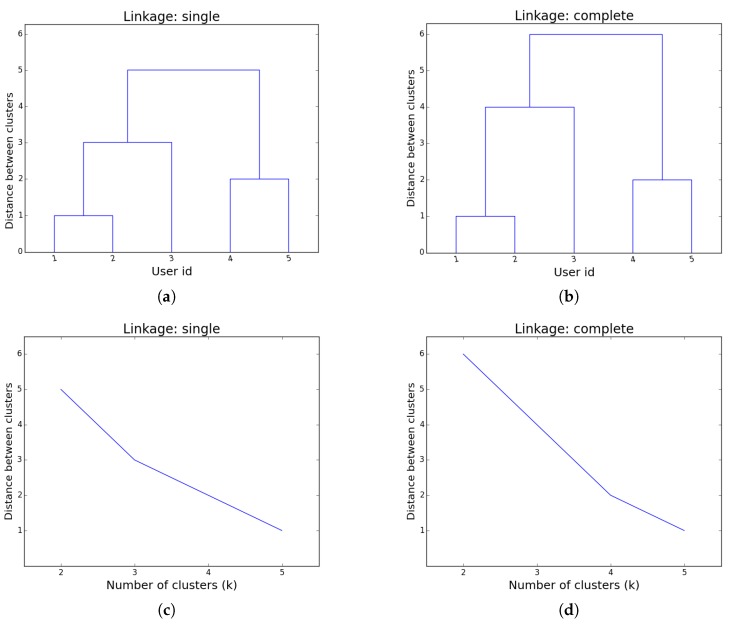
Hierarchical clustering for the group of users in Example 2, (**a**) dendogram for single linkage; (**b**) dendogram for complete linkage; (**c**) number of clusters against distance between clusters for single linkage; (**d**) number of clusters against distance between clusters for complete linkage.

*and assume we want to perform hierarchical clustering using the Manhattan distance with two linkage criteria: single and complete. The hierarchy of clusters obtained for both linkage methods happens to be the same (see Figure 2a,b): $\left(\left(\left(u_{1},u_{2}\right),u_{3}\right),\left(u_{4},u_{5}\right)\right)$, but if we were to plot the number of clusters k against the distance between the last two clusters merged when transitioning from the best k-clustering to the best k−1 clustering (according to the particular linkage method chosen), one would get a contradictory situation. Namely, although we have the exact same clustering, the change in tendency is when k=3 if single linkage is used, and k=4 when using complete linkage (see Figure 2c,d).*

Therefore, we propose a uniform way of deciding the best *k*. Given a set $\left\{u_{1},\ldots ,u_{l}\right\}$ of users and a particular partition $G_{1},\ldots ,G_{k}$ of this set into *k* clusters, we compute the $LM^{2}$-dimensional vectors $A_{u_{1}},\ldots ,A_{u_{l}}$ as per *Model a* (see Equation (2)) and then we evaluate the *within-cluster sum of squares* (WCSS) error:

(10) $$WCSS=\sum_{j=1}^{k}\sum_{u\in G_{j}}{∥A_{u}-q_{j}∥}^{2}$$

where $q_{j}=\frac{1}{|G_{j}|}\sum_{u\in G_{j}}A_{u}$ is the centroid of the points representing users in $G_{j}$.

Note that, in principle, *Model b* could also be used to represent users, but *Model a* offers a finer description. Moreover, partitioning-based clustering methods allow us to find groups of similar users that optimize the WCSS error. In particular, the *k*-means algorithm, a heuristic method commonly employed that converges quickly to a local optimum (see [21]), was used in a related study [22]. Nevertheless, there are several reasons for which hierarchical clustering is a better option in this case:

- *k*-means is very sensitive to outlier examples (such examples can affect the mean by a lot),
- *k*-means works well only for round-shaped, and roughly equal sizes/density clusters (and performs badly if the clusters have non-convex shapes),
- in hierarchical clustering, we do not actually need the value of *k*; instead, the clusterings obtained for different values of *k* can be “visualized” (via the dendrogram) even for points in higher dimensional spaces (helping decide where to cut).

Once *k* is chosen and the groups of similar users are identified, the next step is to find a model that describes the profile of each group of users. Note that groups of users that contain less than 10% of the population are considered *outliers* (the threshold can be modified depending on the application). We propose to train a weighted automaton for each group of users (representing a cluster). We define the *group profile* of cluster *G* as the weighted automaton $M_{G}:={\left\{W_{c}^{G}\right\}}_{c\in C}$ over alphabet C with |A| states, in which the weights matrices are defined as follows.

(11) $$W_{c}^{G}\left(a,a^{\prime }\right)=\frac{\sum_{u_{k}\in G}W_{c}^{k}\left(a,a^{\prime }\right)}{\sum_{c^{\prime }\in C}\sum_{a^{{}^{\prime \prime }}\in A}\sum_{u_{k}\in G}W_{c^{\prime }}^{k}\left(a,a^{{}^{\prime \prime }}\right)},\;\mathrm{for}\mathrm{all}\;c\in C\;\mathrm{and}\;a,a^{\prime }\in A$$

Initial and final probabilities can be similarly defined, but they are not relevant in this case so we omit giving explicit formulas. According to [23], the previous automaton maximizes the likelihood of the observations.

## 6. Case Study: Collaborative Sports Betting

Our proposal was applied to a case study in which thirty users interacted with a mobile groupware application that supports sports betting. These thirty users were randomly grouped in ten groups of three members. They were requested to collaboratively make five bets. The interactions of the users with the application were stored in a log repository.

**Figure 3:**
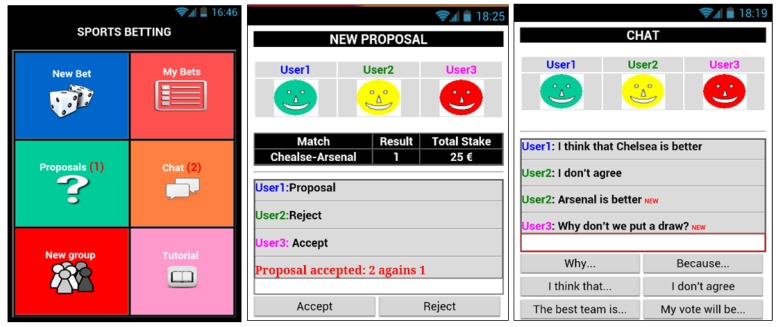
User interface of the groupware system. Main user interface (**left**), chat tool (**center**), voting panel (**right**).

The process of creating a bet is made up of three main steps. First, a user proposes a bet to the other members of the group. This proposal includes a result of a sport event and an amount of money. Second, the members of the group use a chat tool (see Figure 3, right) to analyze the result and the stake of the bet. Third, the members of the group use a voting panel (see Figure 3-center) to accept or reject the bet that has been proposed. Figure 3, left, shows the main user interface of this application with its six panels that allows the user to: propose a new bet to the other members of the group, see the state of the bets or proposals made previously, use a chat to discuss about a proposal, and create a new group of users or see a tutorial that explains how to use the application. The appendix of this paper includes a specification of the actions supported by the application.

We have set L=2 and discretized all time durations into two intervals. The precise intervals are (time is expressed in seconds): $c_{1}=\left[0,104\right],c_{2}=\left[107,5251\right]$, that is, $c_{1}$ corresponds to *short* actions (that take less than one minute and three quarters), and $c_{2}$ corresponds to *long* actions (longer than one minute and three quarters). The threshold is set such that 50% of all possible durations are *short* and the other 50% are *long*.

For each of the three linkage criteria, we generated all possible clusterings (each clustering containing *k* groups, with *k* in {2, …, 30}) for all the models listed in Table 1, each of them with the corresponding distances. This accounts for a total of 1131=(5×3+5×3+3×3)×29 distinct ways of grouping the same set of users, albeit many of them give the same output. In a real life setting, one should be able to choose a priory which model and which measure of similarity is the most appropriate for the application under study. Nevertheless, here we perform an a posteriori analysis, which allows us to select the best combination for our case study.

First of all, we turned our attention to the linkage criteria and we observed the following:

- The single-link or average-link clustering methods detect many outliers before starting to output reasonable sized groups. We report below (see Table 2) the values of *k* at which a second group of at least three users is identified.

- Out of the total of 464=2+3+…+30 groups created for different values of *k*, only a very few of them were proper (having at least 2 members) in the case of average linkage and even fewer for the single linkage criteria. The number of proper groups is presented in Table 3.

Therefore, we concluded that the complete-link clustering methods usually produce more compact clusters and more useful hierarchies than the other two clustering methods.

**Figure 4:**
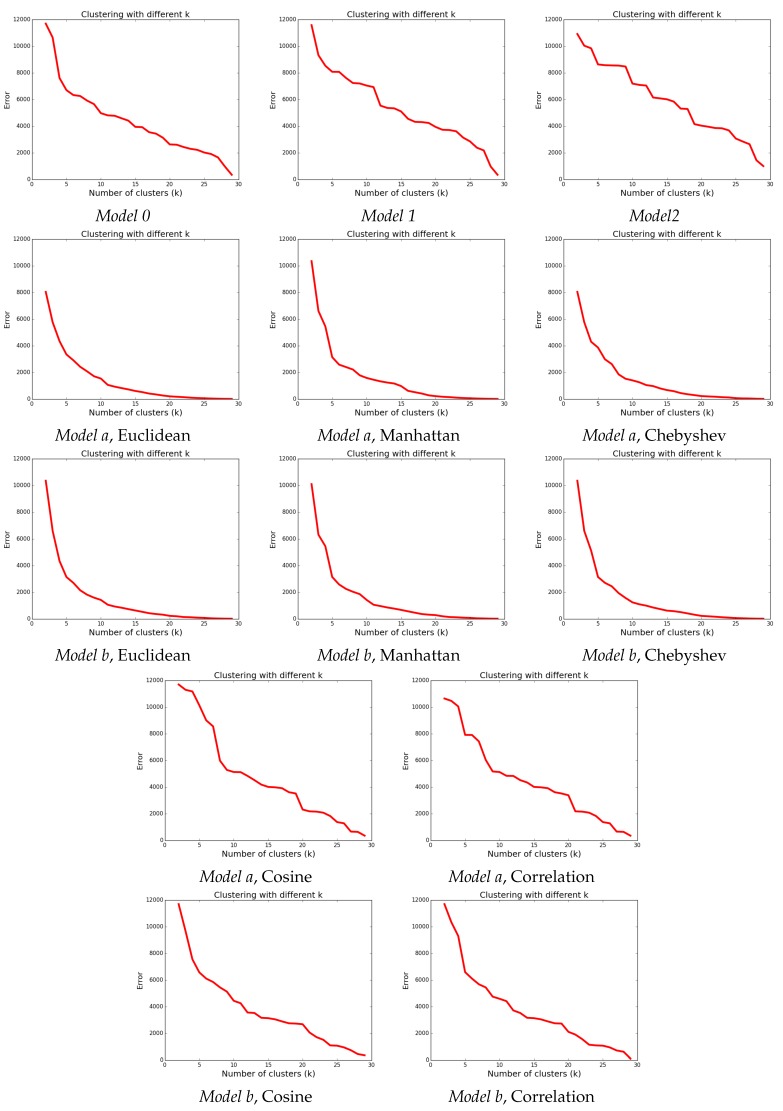
Distance against number of clusters for the complete linkage criteria.

Once chosen the type of linkage, we had to determine which of the five models should be used, and which is the most appropriate measure of similarity between users. As mentioned in Section 5, we plotted the WCSS error against the size of the clustering. The obtained graphics are illustrated in Figure 4.

Inspecting the plots, we can see that for our case study, the three Minkowski type metrics are better: there is a clear steep for small values of *k*, followed by a smooth descent after the “elbow” point. Also, in almost all models, k=5 seems to be an inflection point. And between Euclidean, Manhattan and Chebyshev, we choose Manhattan (with k=5) because, in comparison with the two other distances, Manhattan provides the highest number of proper groups and the lowest *k* at which a second group of at least three users is identified. Note that for the chosen *k*, *Model a* and *Model b* give the same clusterings (this is not true though for k=2 or k=3).

**Figure 5:**
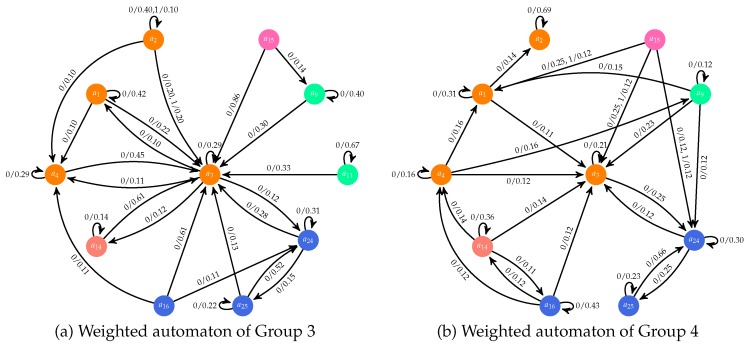
Weighted automata for two profiles; colors indicate the panel to which a given action belongs: orange for Chat, blue for New Bet, green for Proposals, pink for Tutorial and salmon for My bets.

The weighted finite automaton generated for each of the two bigger groups identified with this metric are presented in Figure 5 (the other three groups contained at most two users each, and their characteristics are described in Table 4). A description for each action is provided in the Appendix. A transition from a state $a_{p}$ to a state $a_{r}$ labeled i/x has to be interpreted as $W_{c_{i}}^{G}\left(a_{p},a_{r}\right)=x$. Note that for a better readability, transitions with weights less than 0.1 are omitted.

These automata provide information used to generate a set of rules that enables us to build a description in natural language of the main characteristics of each user profile. Thus, this methodology allows us to build expert system that take as input log files with the users’ interactions and generate as output a textual description of the main user profiles. The following five rules are applied for this purpose:

- Rule 1: If |G|<10%*l for a cluster G→*The members of G can be considered as outliers*.
- Rule 2: If $\exists a,a^{\prime }$ such that $W_{c_{2}}^{G}\left(a,a^{\prime }\right)>0.10\to$*The members of G have used the system in an asynchronous context*.
- Rule 3: If $\exists a,a^{\prime }$ such that $W_{c_{1}}^{G}\left(a,a^{\prime }\right)>0.10\to$*The members of G have used the system in a synchronous context*.
- Rule 4: If $\exists a\in A^{\prime }$ such that $W_{c}^{G}\left(a,a^{\prime }\right)=W_{c}^{G}\left(a^{\prime },a\right)=0,\forall c\in C,\forall a^{\prime }\in A$→*During the interaction process, the members of G never used action a of panel $A^{\prime }$*.
- Rule 5: If $\exists A^{\prime }\subseteq A$ such that $W_{c}^{G}\left(a,a^{\prime }\right)=W_{c}^{G}\left(a^{\prime },a\right)=0,\forall c\in C,\forall a^{\prime }\in A^{\prime }and\forall a\in A$→*During the interaction process, the members of G never used panel $A^{\prime }$*.

Table 4 includes a textual description of the five profiles generated in this case study by means of these five rules. We can observe that the application was often used in a synchronous way. Moreover, we can observe that most users never perform some of the actions supported by the voting panel and some users (groups 2 and 4) access this panel but they do not use all its actions. By simply checking the generated weighted automata, evaluators of the system can easily detect that the actions of accept and reject are very seldom used. The users of the third group accept some proposal of other partners to generate new bets in the list of active bets. However, these users use the chat to answer the proposal of bets that they reject. This illustrates how the automaton and the textual information enable designers to understand the natural user behavior and adapt the system to them. Previous work [3] identified situations in which the users only use some of the actions supported by a specific panel. This new methodological contribution enables us to generate also profiles of users who never use some panel of the interactive system.

Note that there are some actions that are never used by any of the users (actions $a_{12}$ and $a_{13}$ of the *proposals* panel and actions $a_{5}-a_{8}$ of the *chat* panel. These are included separately in the final report and are ignored in the description of the user profiles since they are not specific to any of the five identified profiles.

## 7. Conclusions

We have proposed a methodology for the automatic generation of user interaction models in interactive systems that combines unsupervised and supervised learning. We record the logs of the users in the system in the form of traces and, after a pre-processing phase consisting of describing actions and durations, we use these trajectories (either directly or by compressing them into feature vectors) as input to (various) agglomerative hierarchical clustering algorithms (unsupervised phase) in order to obtain groups of users by similarity of the empirical distributions of actions, durations and transitions. Once this process is completed, we model each group of users by means of a weighted automaton (supervised phase). This finite state machine is what we call a user profile. User profiles, as intended in this paper, constitute a low-level representation of user patterns in interactive systems. From the low-level representation we derive a profile in text form using rules that automatically generate a text identifying some features being representative of the group of users under consideration. As future work we plan to apply our technique to inverse software engineering, using the weighted automaton to infer a task model of the interactive system under study that may help the software engineer to improve the system’s design.

## Figures and Tables

**Table 1:** Summary of models used for clustering.

User’s Model	Measure of Similarity	Linkage
	Euclidian	
*Model a*	Manhattan	single
	Chebyshev	complete
*Model b*	Cosine	average
	Correlation	
*Model 0*		simple
*Model 1*	Monte Carlo	complete
*Model 2*		average

**Table 2:** The minimum number of clusters for which a second group of at least three users is identified.

Single/Complete/Average	Euclidean	Manhattan	Chebyshev	Cosine	Correlation	Monte Carlo
*Model a*	never/5/9	never/4/8	14/4/6	7/5/6	7/2/6	-
*Model b*	11/5/6	12/2/6	9/4/6	8/5/6	8/3/6	-
*Model 0*	-	-	-	-	-	never/3/6
*Model 1*	-	-	-	-	-	20/3/6
*Model 2*	-	-	-	-	-	never/2/never

**Table 3:** The total number of proper groups identified.

Single/Complete/Average	Euclidean	Manhattan	Chebyshev	Cosine	Correlation	Monte Carlo
*Model a*	31/61/47	28/67/44	31/49/42	62/127/100	63/138/104	-
*Model b*	43/87/69	35/94/68	43/82/69	57/105/90	61/111/94	-
*Model 0*	-	-	-	-	-	43/107/73
*Model 1*	-	-	-	-	-	42/126/84
*Model 2*	-	-	-	-	-	28/139/39

**Table 4:** Description of the profiles.

Group	Description of Profiles and Rules that Are Activated
Number/	
Number	
of Users	
1 / 1	The members of this group can be considered as outliers (Rule 1). The members of this group have used the system in a synchronous context (Rule 3).
2 / 1	The members of this group can be considered as outliers (Rule 1). The members of this group have used the system in an asynchronous context (Rule 2). The members of this group have used the system in a synchronous context (Rule 3). During the interaction process, the members of this group never used the *voting* panel (Rule 5).
3 / 9	The members of this group have used the system in an asynchronous context (Rule 2). The members of this group have used the system in a synchronous context (Rule 3). During the interaction process the members of this group never used action *reject* of the *voting* panel (Rule 4).
4 / 17	The members of this group have used the system in an asynchronous context (Rule 2). The members of this group have used the system in a synchronous context (Rule 3). During the interaction process the members of this group never used the *voting* panel (Rule 5).
5 / 2	The members of this group can be considered as outliers (Rule 1). The members of this group have used the system in a synchronous context (Rule 3). During the interaction process the members of this group never used action *reject* of the *voting* panel (Rule 4). During the interaction process the members of this group never used the *tutorial* panel (Rule 5).

## Abbreviations

The following abbreviations are used in this manuscript:

CSCW	computer-supported collaborative work
WCSS	within-cluster sum of squares
HCI	human–computer interaction

## Appendix A

The following table describes the semantics of the actions of the interactive groupware system used for the experimentation. The actions supported by the tool to generate groups of users are omitted because they were managed by the evaluators and the users of the system never performed these actions.

**Table A1:** Actions of the groupware system.

Identifier	Panel	Description of the Action
$a_{1}$	Chat	Access to this space
$a_{2}$	Chat	Send a “fre” message
$a_{3}$	Chat	Send a “Why...” message
$a_{4}$	Chat	Send a “Because” message
$a_{5}$	Chat	Send a “I think that” message
$a_{6}$	Chat	Send a “I don’t agree” message
$a_{7}$	Chat	Send a “The beast team is” message
$a_{8}$	Chat	Send a “My vote will be” message
$a_{9}$	Proposals	Access to this space
$a_{10}$	Proposals	Reject a proposal
$a_{11}$	Proposals	Accept a proposal
$a_{12}$	Proposals	A proposal was accepted by the group
$a_{13}$	Proposals	A proposal was rejected by the group
$a_{14}$	My Bets	Access to this space
$a_{15}$	Tutorial	Access to this space
$a_{16}$	New Bet	See sport events
$a_{24}$	New Bet	Access to this space
$a_{25}$	New Bet	Send a proposal

## References

[1] Grudin J. (1994). Computer-Supported Cooperative Work: History and Focus. Computer.

[2] Cruz A., Correia A., Paredes H., Fonseca B., Morgado L., Martins P., Herskovic V., Hoppe H.U., Jansen M., Ziegler J. (2012). Towards an Overarching Classification Model of CSCW and Groupware: A Socio-technical Perspective. Proceedings of the 18th International Conference on Collaboration and Technology.

[3] Tirnăucă C., Duque R., Montaña J.L., García C.R., Caballero-Gil P., Burmester M., Quesada-Arencibia A. (2016). Automatic Generation of User Interaction Models. Proceedings of the Ubiquitous Computing and Ambient Intelligence—10th International Conference, UCAmI 2016, San Bartolomé de Tirajana.

[4] Kobsa A. (2001). Generic User Modeling Systems. User Model. User-Adapt. Interact..

[5] Kobsa A., Pohl W. (1995). The User Modeling Shell System BGP-MS. User Model. User-Adapt. Interact..

[6] Skillen K., Chen L., Nugent C.D., Donnelly M.P., Burns W., Solheim I., Bravo J., López-de-Ipiña D., Moya F. (2012). Ontological User Profile Modeling for Context-Aware Application Personalization. Proceedings of the Ubiquitous Computing and Ambient Intelligence—6th International Conference, Vitoria-Gasteiz.

[7] Castillejo E., Almeida A., de Ipiña D.L. (2014). Ontology-Based Model for Supporting Dynamic and Adaptive User Interfaces. Int. J. Hum.–Comput. Interact..

[8] Razmerita L., Angehrn A.A., Maedche A., Brusilovsky P., Corbett A.T., de Rosis F. (2003). Ontology-Based User Modeling for Knowledge Management Systems. Proceedings of the User Modeling 2003 9th International Conference.

[9] Hatala M., Wakkary R. (2005). Ontology-Based User Modeling in an Augmented Audio Reality System for Museums. User Model. User-Adapt. Interact..

[10] Castillejo E., Almeida A., López-de-Ipiña D., Chen L. (2014). Modeling Users, Context and Devices for Ambient Assisted Living Environments. Sensors.

[11] Suárez Torrente M.C., Martínez Prieto A.B., Alvarez Gutiérrez D., Alva de Sagastegui M.E. (2013). Sirius: A heuristic-based framework for measuring web usability adapted to the type of website. J. Syst. Softw..

[12] Molina A.I., Gallardo J., Redondo M.A., Bravo C. (2014). Evaluating the Awareness Support of COLLECE, a Collaborative Programming Tool. Proceedings of the Interacción ’14 XV International Conference on Human Computer Interaction, Puerto de la Cruz.

[13] Neale D.C., Carroll J.M., Rosson M.B. (2004). Evaluating Computer-supported Cooperative Work: Models and Frameworks. Proceedings of the CSCW ’04 2004 ACM Conference on Computer Supported Cooperative Work.

[14] Van der Aalst W.M.P. (2007). Exploring the CSCW Spectrum Using Process Mining. Adv. Eng. Inform..

[15] Thaler T., Plödereder E., Grunske L., Schneider E., Ull D. (2014). Towards Usability Mining. Proceedings of the 44. Jahrestagung der Gesellschaft für Informatik, Informatik 2014, Big Data—Komplexität meistern.

[16] Bravo C., Redondo M.A., Verdejo M.F., Ortega M. (2008). A framework for process-solution analysis in collaborative learning environments. Int. J. Hum.-Comput. Stud..

[17] Culik K., Karhumaki U. (1994). Finite Automata Computing Real Functions. SIAM J. Comput..

[18] Tirnăucă C., Montaña J.L., Ontañón S., Gonzalez A.J., Pardo L.M. (2016). Behavioral Modeling Based on Probabilistic Finite Automata: An Empirical Study. Sensors.

[19] Ontañón S., Montaña J.L., González A.J. (2014). A Dynamic-Bayesian Network framework for modeling and evaluating learning from observation. Expert Syst. Appl..

[20] Rokach L., Maimon O., Rokach L. (2010). A survey of Clustering Algorithms. Data Mining and Knowledge Discovery Handbook.

[21] Everitt B.S., Landau S., Leese M., Stahl D. (2011). Cluster Analysis.

[22] Duque R., Montaña J.L., Tîrnăucă C. Mining the dataflow in interactive systems: Automatic generation of user behavior patterns. Proceedings of the Actas de la XVII Conferencia de la Asociación Española para la Inteligencia Artificial (CAEPIA ’16), Parte IV.-VIII Simposio Teoría y Aplicaciones de Minería de Datos (TAMIDA ’16).

[23] Dupont P., Denis F., Esposito Y. (2005). Links between probabilistic automata and hidden Markov models: Probability distributions, learning models and induction algorithms. Pattern Recognit..

